# Expression profiling reveals transcriptional regulation by Fbxw7/mTOR pathway in radiation-induced mouse thymic lymphomas

**DOI:** 10.18632/oncotarget.6328

**Published:** 2015-11-02

**Authors:** Antoine M. Snijders, Yueyong Liu, Li Su, Yurong Huang, Jian-Hua Mao

**Affiliations:** ^1^ Life Sciences Division, Lawrence Berkeley National Laboratory, Berkeley, CA, USA; ^2^ Department of Pathology, Beth Israel Deaconess Medical Center, Harvard Medical School, Boston, MA, USA

**Keywords:** thymic lymphoma, FBXW7, mTOR, rapamycin, radiation

## Abstract

The tumor suppressor gene *FBXW7* is deleted and mutated in many different types of human cancers. FBXW7 primarily exerts its tumor suppressor activity by ubiquitinating different oncoproteins including mTOR. Here we used gene transcript profiling to gain a deeper understanding of the role of *FBXW7* in tumor development and to determine the influence of mTOR inhibition by rapamycin on tumor transcriptome and biological functions. In comparison to tumors from p53 single heterozygous (p53+/−) mice, we find that radiation-induced thymic lymphomas from *Fbxw7*/*p53* double heterozygous (*Fbxw7+/−p53+/−*) mice show significant deregulation of cholesterol metabolic processes independent of rapamycin treatment, while cell cycle related genes were upregulated in tumors from placebo treated *Fbxw7+/−p53+/−* mice, but not in tumors from rapamycin treated *Fbxw7+/−p53+/−* mice. On the other hand, tumors from rapamycin treated *Fbxw7+/−p53+/−* mice were enriched for genes involved in the integrated stress response, an adaptive mechanism to survive in stressful environments. Finally, we demonstrated that the *Fbxw7* gene signatures identified in mouse tumors significantly overlap with *FBXW7* co-expressed genes in human cancers. Importantly these common *FBXW7* gene signatures between mouse and human are predictive for disease-free survival in human colon, breast and lung adenocarcinoma cancer patients. These results provide novel insights into the role of *FBXW7* in tumor development and have identified a number of potential targets for therapeutic intervention.

## INTRODUCTION

The human tumor suppressor gene *FBXW7* encodes an F-box protein, mutated and deleted in cancers from a wide spectrum of human tissues, such as bile duct [1], blood [2–5], bone [6], brain [7, 8], breast [9], colon [10], endometrium [11], stomach [12], lung [13], ovary [14], pancreas [15], and prostate [16]. The overall point mutation frequency of *FBXW7* in human cancers is approximately 6% [1]. In the mouse, homozygous deletion of the *Fbxw7* gene leads to embryonic lethality, but heterozygous mice develop normally [17, 18]. Although they do not develop spontaneous tumors, radiation exposure gives rise to different types of tumors, including a range of epithelial cancers, albeit at low frequency. Mice that carry inactivated alleles of both *Fbxw7* and *p53* show acceleration of tumor development after radiation exposure [19]. Mechanistically, it has been shown that FBXW7 is essential for the ubiquitination of different oncoproteins, including c-Myc [20, 21], c-Jun [22], CCNE [23–25], different members of the Notch family [26–28], Aurora-A [19, 29], and mTOR [30].

How does the decrease in FBXW7 function result in tumor development? Deletion or mutation of the *FBXW7* gene may result in impaired degradation of multiple targets and their consequent accumulation, which may cooperatively contribute to tumor development. Our previous studies using mouse models showed that temporal pharmacological inhibition of the mTOR pathway was sufficient to suppress the tumor development contributed by *Fbxw7* loss, suggesting that the Fbxw7-mTOR pathway plays a major role in this radiation-induced carcinogenesis mouse model [31]. In this study, we used gene profiling technology to elucidate the signaling pathways by which Fbxw7 is involved, which could lead to identification of new targets for therapeutic intervention.

## RESULTS

### Significant deregulation of cholesterol metabolic processes in tumors from *Fbxw7*/*p53* double heterozygous mice

**Figure 1:**
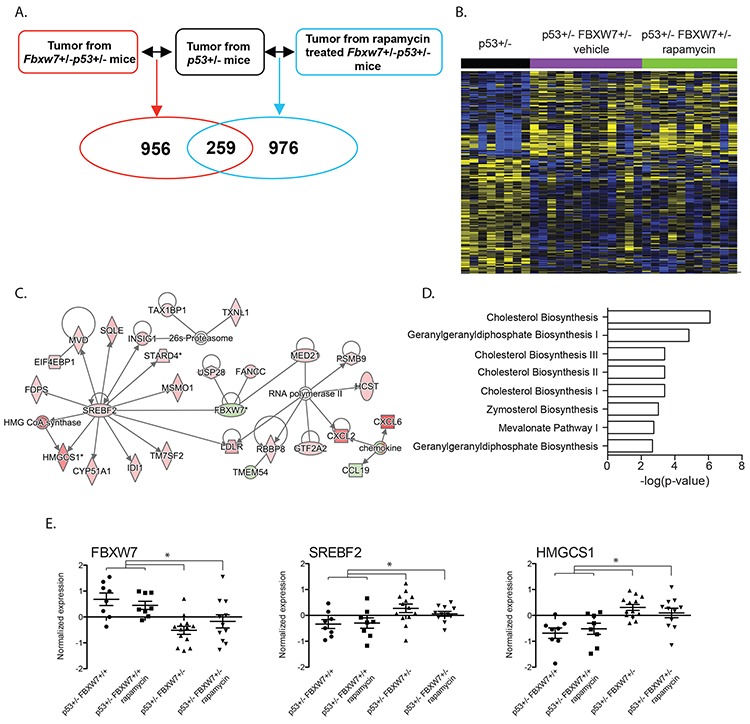
Significant transcriptional deregulation in *Fbxw7* heterozygous thymic lymphomas **A.** Gene transcript levels in thymic lymphomas from vehicle and rapamycin treated *Fbxw7*+/−*p53*+/− mice were compared to these in thymic lymphomas from *p53*+/− mice. **B.** Unsupervised hierarchical clustering of 259 genes differentially expressed in tumors isolated from vehicle and rapamycin treated *Fbxw7*+/−*p53*+/− mice compared to *p53*+/− mice (increased expression indicated in yellow; decreased expression indicated in blue). **C.** Gene interaction network of 259 overlapping genes. **D.** Pathways significantly associated with 259 overlapping genes. **E.** Normalized expression of *Fbxw7*, *Srebf2* and *Hmgcs1* in radiation induced thymic lymphomas of vehicle or rapamycin treated *p53*+/−,and vehicle or rapamycin treated *Fbxw7*+/−*p53*+/− mice.

We previously showed that loss of a single copy of the tumor suppressor *Fbxw7* significantly reduced radiation-induced tumor latency in *p53*+/− mice [19]. Among its functions, depletion of *FBXW7* alleviates the inhibitory effect on mTOR signaling resulting in activation of the mTOR pathway. Our previous study showed that temporal inhibition of mTOR by rapamycin significantly delayed tumor development in *Fbxw7*/*p53* double heterozygous (*Fbxw7*+/−*p53*+/−) mice [31]. To determine the molecular mechanisms associated with these observations we transcriptionally profiled radiation-induced thymic lymphomas from rapamycin (*n* = 11) and vehicle (*n* = 13) treated *Fbxw7*+/−*p53*+/− mice or from vehicle treated p53 single heterozygous (*p53*+/−) mice (*n* = 8) (Figure 1A; Supplementary Table S1 for experimental details). In comparison to transcriptome of thymic lymphomas from *p53*+/− mice, we found 1215 and 1235 mapped genes differentially expressed in thymic lymphomas from vehicle and rapamycin treated *Fbxw7*+/−*p53*+/− mice respectively (fold-change 1.3; *p* < 0.05, Figure 1A), with 259 overlapping genes that were differentially expressed independent of rapamycin treatment (Figure 1A and 1B). We next computationally mapped the overlapping 259 transcripts to biological functions, pathways and upstream transcriptional regulators using Ingenuity Pathway Analysis (IPA). As expected, gene interaction network analyses confirmed downregulation of *Fbxw7* expression in *Fbxw7*+/− mice (Figure 1C and 1E). Interestingly, we also observed significant upregulation of SREBF2 (Figure 1C and 1E). We validated these findings using an independent tumor set from rapamycin treated p53 single heterozygous (*p53*+/−) mice (*n* = 8). When we combined the transcript data from this tumor set with the transcript data of thymic lymphomas from vehicle treated *p53*+/− mice (*n* = 8) significant fold-changes were found for FBXW7, SREBF2 and HMGCS1 (Figure 1E) confirming our results. Consistent with this observation, we found significant enrichment of genes regulated by SREBF1 and 2 (Table 1, Supplementary Table S2; *p* < 7.22E-08) and significant enrichment of pathways involved in cholesterol metabolism (Figure 1D; Supplementary Table S2). SREBF1 and SREBF2 bind sterol regulatory sequences in promoters of genes involved in sterol metabolic processes. Our data suggests that loss of *FBXW7* results in up-regulation of cholesterol and sterol metabolism, independent of the mTOR pathway. Consistent with this finding are previous reports linking FBXW7 with SREBF and a role for *FBXW7* in regulating lipid metabolism in the mouse liver [32, 33].

**Table 1:** Upstream transcriptional regulators significantly associated with genes in thymic lymphomas of vehicle and rapamycin treated mice

Upstream Regulators	*Fbxw7*+/−*p53*+/− vehicle (unique)	*Fbxw7*+/−*p53*+/− (overlapping gene set)	*Fbxw7*+/−*p53*+/− rapamycin (unique)	*p53*+/− rapamycin
TP53	3.27E–06			
HTT	3.40E–06			
GATA1	8.66E–06			
TP73	1.30E–05			
CCND1	1.83E–05			
YY1	2.33E–05			
HNF1A	2.34E–05			
SIM1	7.44E–05			
CREM	1.02E–04	1.89E–03		
EGR2	1.22E–04			
BCL11A	1.40E–04			
KLF13	1.72E–04			
ARNT2	1.77E–04			
CDKN2A	1.89E–04			
BRCA1	2.28E–04			
HIPK2	4.77E–04			
STAT3	4.94E–04			
MAFK	5.08E–04			
FOXP3	7.24E–04			
NR3C1	7.64E–04			
NFATC2	7.68E–04			
PPARA	9.77E–04	1.48E–05		
BACH2	1.06E–03			
EGR1	1.12E–03	6.43E–04		
KDM1A	1.21E–03			
JUND	1.31E–03			
IRF4	1.65E–03			
CREBBP	1.67E–03			
MMP14	1.71E–03			
NR3C2	1.87E–03			
RB1	2.24E–03			
STAT6	2.49E–03			
TCF3	2.88E–03			
TIAL1	3.20E–03			
ID2	3.23E–03			
CEBPB	3.37E–03			
FOXO1	3.57E–03			
HIST2H3C	3.62E–03			
BATF	3.75E–03			
REL	4.74E–03			
MECP2	4.80E–03			
SREBF2		9.01E–13	2.52E–03	
SREBF1		7.22E–08		
HNF4A		1.69E–04	7.53E–08	
E2F6		1.72E–04		
CREB1		2.62E–04		
SIRT2		3.46E–04		
NC2		4.57E–04		
FOXO4		4.58E–04		
ESRRA		7.51E–04		
PPARGC1B		7.74E–04		
PPARGC1A		2.21E–03		
PML		3.04E–03		
AR		4.54E–03		
E2F		4.79E–03		
ATF4			1.55E–04	
RUVBL1			2.32E–03	
TP53			2.57E–03	
DDIT3			3.00E–03	
PTBP1			3.48E–03	
HIF1A			4.14E–03	
RORA				2.49E–03
MKL2				4.70E–03

### Upregulation of cell cycle related genes in tumors from *Fbxw7*+/−*p53*+/− mice

**Figure 2:**
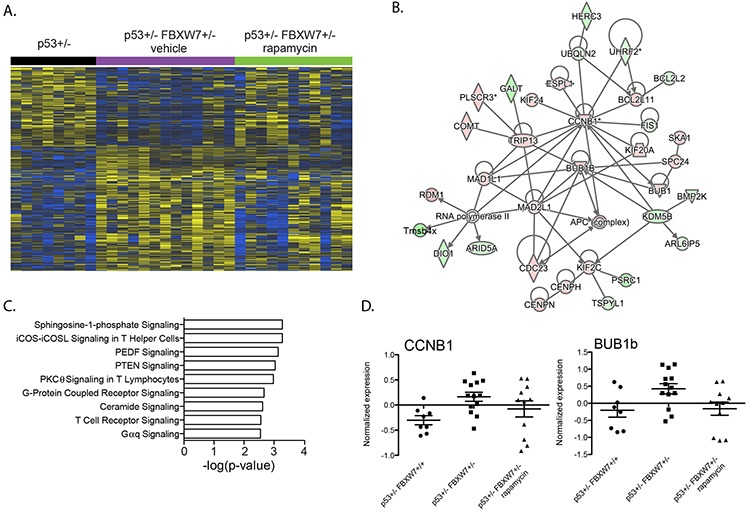
Enrichment of cell cycle related genes in *Fbxw7* heterozygous thymic lymphomas **A.** Unsupervised hierarchical clustering of 956 genes differentially expressed in thymic lymphomas of vehicle treated *Fbxw7*+/−*p53*+/− mice compared to thymic lymphomas of *p53*+/− mice (increased expression indicated in yellow; decreased expression indicated in blue). **B.** Gene interaction network of 956 genes enriched with cell cycle related genes. **C.** Pathways significantly associated with 956 gene set. **D.** Normalized expression of *Ccnb1* and *Bub1b* in radiation induced thymic lymphomas of *p53*+/− and vehicle or rapamycin treated *Fbxw7*+/−*p53*+/− mice.

We next analyzed tumor transcript level changes that were unique to the vehicle treated *Fbxw7*+/−*p53*+/− mice (956 genes; Figure 1A). Network analyses showed significant enrichment of cell cycle related genes (Figure 2; *p* < 3.79E-02) consistent with the tumor suppressor functions of *Fbxw7* in cellular growth and division pathways. Interestingly, we observed significant enrichment of spingosine-1-phosphate (S1P) signaling (Figure 2C; Supplementary Table S2). Cross-talk between the S1P and mTOR pathways has been described and our data suggests a role for FBXW7 in the S1P-mTOR axis [34, 35]. Two representative examples of genes upregulated in vehicle treated *Fbxw7*+/−*p53*+/− mice, but not rapamycin treated *Fbxw7*+/−*p53*+/− mice are shown in Figure 2D. Significant upstream transcriptional regulators include TP53, TP73 and CCND1 (Table 1, Supplementary Table S2; *p* < 1.83E-05). These results indicate that radiation induced tumors that arise in a *Fbxw7*+/−*p53*+/− background exhibit decreased latency mediated at least in part through increased cell proliferation mechanisms, and temporal rapamycin treatment delayed tumor development through the inhibition of cell proliferation pathways. We next addressed how rapamycin treatment after radiation exposure can alter tumor transcript profiles.

### Upregulation of integrated stress response genes in the tumors from rapamycin treated *Fbxw7*+/−*p53*+/− mice

**Figure 3:**
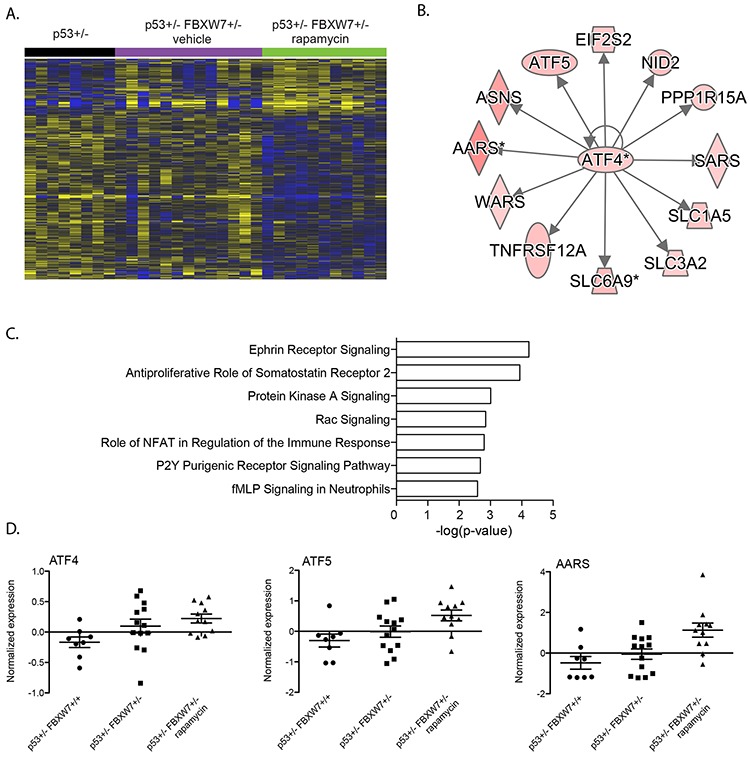
ATF4 stress response genes upregulated in *Fbxw7* heterozygous thymic lymphoma after rapamycin treatment **A.** Unsupervised hierarchical clustering of 976 genes differentially expressed in thymic lymphomas of rapamcyin treated *Fbxw7*+/−*p53*+/− mice compared to thymic lymphomas of *p53*+/− mice (increased expression indicated in yellow; decreased expression indicated in blue). **B.** Upstream transcriptional regulator analyses of 976 genes differentially expressed in rapamycin treated mice revealed a significant association with ATF4. **C.** Pathways significantly associated with 976 gene set. **D.** Normalized expression of *Atf4*, *Atf5* and in radiation induced thymic lymphomas of *p53*+/− and vehicle or rapamycin treated *Fbxw7*+/−*p53*+/− mice.

We analyzed transcript profiles of tumors isolated from rapamycin treated *Fbxw7*+/−*p53*+/− mice. We found 976 transcripts differentially expressed in rapamycin treated *Fbxw7*+/−*p53*+/− thymic lymphomas when compared to *p53*+/− tumors (Figure 1A). We observed that the cell cycle enrichment observed in vehicle treated mice was not present in tumors that arose in rapamycin treated mice during tumor development. In addition, we observed a shift in upstream transcriptional regulators (Table 1, Supplementary Table S2). We observed significant enrichment of genes regulated by ATF4 and DDIT3 in tumors from rapamycin treated mice (Figure 3; Table 1, Supplementary Table S2). These regulators were not associated with transcript levels in vehicle treated mice. ATF4 and DDIT3 upregulation are indicative of activation of the integrated stress response to accumulation of unfolded proteins in the endoplasmic reticulum, amino acid starvation or oxidants. To determine whether this response was dependent on loss of *Fbxw7*, we compared transcript profiles of tumors from rapamycin treated *Fbxw7*+/−*p53*+/− mice and rapamycin treated *p53*+/− mice. We found that the transcriptional regulators ATF4 and DDIT3 were not significantly associated with transcript levels in tumors of rapamycin treated *p53*+/− mice (Table 1, Supplementary Table S2) indicating that the ATF4/DDIT3 regulated transcript response is dependent on rapamycin exposure in an environment depleted of *Fbxw7*.

### *FBXW7* gene signatures are associated with clinical outcome in human cancer

**Figure 4:**
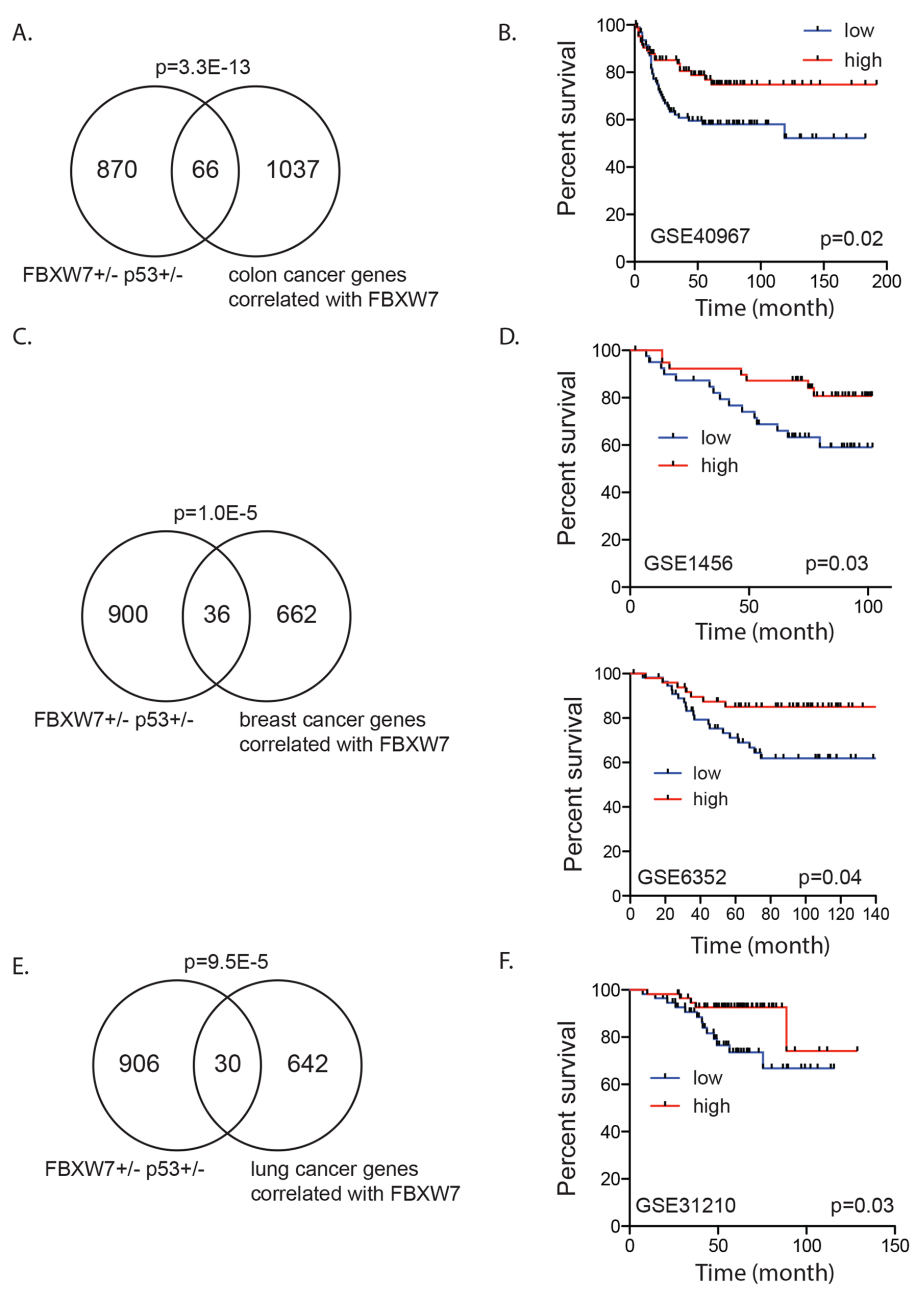
*Fbxw7* correlated gene expression signature associated with disease free survival in human cancer patients **A.** Significant overlap between genes deregulated in thymic lymphomas of *Fbxw7*+/−*p53*+/− mice compared to *p53+/−* mice and genes positively correlated with FBXW7 (Pearson correlation > 0.5) in human colorectal adenocarcinoma (*p*-value of enrichment 3.3E-13). **B.** Increased expression of overlapping gene signature is associated with increased disease free survival in human colon cancer patients (GSE40967; *p* = 0.02). **C.** Significant overlap between genes deregulated in thymic lymphomas of *Fbxw7*+/−*p53*+/− mice compared to *p53+/−* mice and genes positively correlated with FBXW7 (Pearson correlation > 0.2) in human breast cancer patients (*p*-value of enrichment 1.0E-05). **D.** Increased expression of overlapping gene signature is associated with increased disease free survival in human breast cancer patients (GSE1456; *p* = 0.03 and GSE6352; *p* = 0.04). **E.** Significant overlap between genes deregulated in thymic lymphomas of *Fbxw7*+/−*p53*+/− mice compared to *p53+/−* mice and genes positively correlated with FBXW7 (Pearson correlation > 0.2) in human lung cancer patients (*p*-value of enrichment 9.5E-05). **F.** Increased expression of overlapping gene signature is associated with increased disease free survival in human lung cancer patients (GSE31210; *p* = 0.03).

A cross-cancer analysis (cBioPortal, The Cancer Genome Atlas) revealed that *FBXW7* is frequently altered in many cancer types including colon, breast and lung. Loss of one copy of *FBXW7* accounted for the majority of alterations. We investigated if genes whose expression in human tumors correlated with *FBXW7* expression showed overlap with the genes deregulated in the *Fbxw7*+/−*p53*+/− mouse thymic lymphomas. Using cBioPortal, we generated three lists consisting of 1103, 698 and 672 genes of which expression levels positively correlated with *FBXW7* expression in human colorectal adenocarcinoma, breast cancer and lung adenocarcinoma, respectively [36, 37]. Interestingly, we observed a very significant overlap between genes that are deregulated in *Fbxw7+/−/p53+/−* mouse tumors and those that are co-expressed with *FBXW7* in colon (66 genes; *p* = 3.3E-13), breast (36 genes; *p* = 1.0E-05) and lung cancer (30 genes; *p* = 9.5E-05), respectively (Figure 4A, 4C, and 4E). Given the role of *FBXW7* as a tumor suppressor gene we predicted that higher expression of these overlapping and *FBXW7* positively correlated genes would have a good prognosis for patients. We tested the association of transcript levels for the corresponding *FBXW7* gene signatures in colon, breast and lung cancer patients, for which information on disease-free survival and gene expression was available [38–40]. We defined a score of *FBXW7* gene signature for each patient as the sum of the normalized expression intensities of all genes in each signature. We then examined whether there was a difference in disease free survival for the top 25% of patients with highest and bottom 25% patients with lowest sum expression. For all three tumor types, we observed that patients with a higher score (top 25%) had significantly increased survival compared to patients with a low score (bottom 25%) (Figure 4B, 4D, and 4F). Taken together, our results reinforce the role of *FBXW7* as a tumor suppressor and indicate that FBXW7 is a viable target for therapeutic intervention.

## DISCUSSION

In a mouse model of radiation-induced carcinogenesis we showed that loss of a single copy of the tumor suppressor *FBXW7* significantly reduced tumor latency and that temporal inhibition of mTOR pathway was sufficient to suppress tumor development contributed by *Fbxw7* loss in this model, suggesting that Fbxw7-mTOR pathway plays a major role. Here we used transcript profiling of radiation induced thymic lymphomas to investigate the molecular signaling pathways that contribute to tumorigenesis in *Fbxw7* heterozygous mice and their dependence on mTOR. We observed significant enrichment of genes regulated by SREBF1 and 2 (sterol regulatory element binding transcription factors 1 and 2; SREBP1, SREBP2). The FBXW7-SREBF axis has been proposed in mouse models of non-alcoholic fatty liver disease [32, 33]. In this study FBXW7 and SREBF1 were negatively correlated consistent with our observation of increased expression of SREBF1 and downstream target genes in thymic lymphomas of FBXW7 heterozygous mice. Phosphorylation of SREBF has been shown to create a recognition site for FBXW7 and subsequent degradation by the multiprotein SCF complex [41]. Our results suggest that loss of FBXW7 results in increased activity of SREBF contributing to tumorigenesis. Interestingly, accumulating evidence has indicated that SREBF1 proteins regulate tumorigenesis [42–45]. In pancreatic cancer, increased expression of SREBF1 was predictive of poor prognosis and depletion of SREBF1 resulted in suppression of tumor growth [46]. Rapamycin treatment of cultured human adipocytes resulted in down-regulation of gene expression of SREBF1 [47] suggesting that the tumor promoting effects of the FBXW7-SREBF axis is dependent on mTOR. Temporal inhibition of mTOR by rapamycin in our mouse model resulted in delayed tumor latency. Our transcript analyses, however, did show the tendency to downregulation of SREBF1 in tumors from *Fbxw7* heterozygous mice treated with rapamycin compared to vehicle control treated tumors (Figure 1D middle panel). Taken together, our results provide further evidence for a role of SREBF proteins in tumorigenesis through loss of *FBXW7* and indicates SREBF as a target for therapeutic intervention in tumors with FBXW7 deregulation.

In *Fbxw7* heterozygous mice temporally treated by rapamycin for ten consecutive weeks after radiation exposure, we observed significant enrichment and upregulation of ATF4 and DDIT3 indicative of activation of the integrated stress response. Numerous challenges to the endoplasmatic reticulum (ER) can cause the accumulation of unfolded proteins in the ER resulting in the unfolded protein response (UPR). Challenges include anoxia, amino acid starvation, glucose deprivation and oxidants. The initial response to this stress is to restore homeostasis promoting cell survival by increasing expression of genes involved in protein folding and degradation of misfolded proteins. When the initial response fails, prolonged ER stress can result in apoptosis. Our study shows upregulation of ATF4, one of the mediating transcription factors of the UPR. Tumors often grow under suboptimal conditions in an environment low in oxygen and nutrients. Thus, it may not be surprising that adaptation to ER stress is a determinant of tumor progression [48, 49]. It should be noted that tumors of *Fbxw7* wild type, but otherwise isogenic mice concurrently treated with rapamycin did not exhibit enrichment of this response suggesting cross-talk between FBXW7 and mTOR and ER stress. Recently, bidirectional cross talk between mTOR and the UPR have been suggested with mTOR functioning both upstream and downstream of ER stress signals [50–52]. Inhibition of mTOR for example increases cell viability during ER stress [53, 54]. Our data suggests that temporal inhibition of mTOR by rapamycin during early tumor development may result in persistent ER stress in tumors that develop long after rapamycin treatment. Further research is needed to clarify the role of FBXW7 in the UPR and tumor development.

## MATERIALS AND METHODS

### Mice, irradiation and rapamycin treatment

All animal experiments were performed at Lawrence Berkeley National Laboratory and the study was carried out in strict accordance with the Guide for the Care and Use of Laboratory Animals of the National Institutes of Health. The animal use protocol was approved by the Animal Welfare and Research Committee of the Lawrence Berkeley National Laboratory. Detailed methods were described previously [31]. Briefly, *p53*+/− and *p53*+/− *Fbxw7*+/− mice were generated by crossing p53−/− mice with *Fbxw7*+/− mice. At 5 weeks of age, mice were exposed whole-body to a single dose of 4 Gy X-ray irradiation. Mice were divided randomly into two groups and treated with rapamycin or placebo [31].

### RNA isolation and transcript profiling

Total RNA quality and quantity were determined using Agilent 2100 Bioanalyzer and NanoDrop ND-1000. Agilent SurePrint G3 Mouse GE 8 × 60 K Microarrays were used according to the manufacturer's protocol (arrays contained 39,430 Entrez gene RNAs and 16,251 lncRNAs). All processes were performed by Ambry Genetics (Aliso Viejo, CA). Microarray data have been deposited at NCBI GEO (accession number: GSE71975).

### Data analysis

Data normalization was performed using GeneSpring GX12.5 (Agilent Technologies). Signal intensities for each probe were normalized to the 75th percentile without baseline transformation. Genes that were differentially expressed in thymic lymphomas between vehicle and rapamycin treated *Fbxw7+/−p53+/−* mice and *p53+/−* mice were identified by the unpaired Student's *t*-test with a *p*-value cut-off of less than 0.05 and a fold change criteria of more than 1.3. Gene lists were annotated with biological functions using Ingenuity Pathway Analysis (IPA), KEGG pathway analysis (http://bioinfo.vanderbilt.edu/webgestalt/) and DAVID GO gene ontology (http://david.abcc.ncifcrf.gov/; *p* ≤ 0.05).

### Human cancer datasets and survival analysis

The list of genes that are co-expressed with *FBXW7* in colon, lung and breast cancer were obtained from the TCGA study at cBioPortal. The overlap between *FBXW7* co-expressed genes and the set of genes deregulated in *Fbxw7+/−/p53+/−* mouse tumors was used to test for association with disease-free survival. Gene expression microarray datasets of colon, lung and breast cancer for which disease-free survival was available were downloaded from the NCBI GEO website. Kaplan-Meier plots were constructed and a long-rank test was used to determine differences among disease free survival according to a score of *FBXW7* gene signature for each patient as the sum of the normalized expression intensities of all genes in each signature.

## SUPPLEMENTARY TABLES






