# Supplementary material for: Expression profiling reveals transcriptional regulation by Fbxw7/mTOR pathway in radiation-induced mouse thymic lymphomas
Source: Oncotarget. 2015 Nov 2;6(42):44794–805. doi: 10.18632/oncotarget.6328 (PMC4792592; doi:10.18632/oncotarget.6328)
Supplement: Supplementary file 1 [file oncotarget-06-44794-s001.pdf]

SUPPLEMENTARY TABLES

Supplementary Table S1: Tumor sample information

Supplementary Table S2: Upstream transcriptional regulators and canonical pathways significantly associated with genes in thymic lymphomas of vehicle and rapamycin treated mice
